# Characterization of avian influenza H9N2 viruses isolated from ostriches (*Struthio camelus*)

**DOI:** 10.1038/s41598-018-20645-1

**Published:** 2018-02-02

**Authors:** Dongdong Wang, Jingjing Wang, Yuhai Bi, Dandan Fan, Hong Liu, Ning Luo, Zongtong Yang, Shouchun Wang, Wenya Chen, Jianlin Wang, Shouzhen Xu, Jiming Chen, Yi Zhang, Yanbo Yin

**Affiliations:** 1 Laboratory of Preventive Veterinary Medicine, College of Animal Science and Veterinary Medicine, Qingdao Agricultural University, Qingdao, 266019 China; 2 China Animal Health and Epidemiology Center, Qingdao, 266032 China; 3 CAS Key Laboratory of Pathogenic Microbiology and Immunology, Institute of Microbiology, Center for Influenza Research and Early-warning (CASCIRE), Chinese Academy of Sciences, Beijing, China; 4 Qingdao Oland-Better Bioengineering Co., LTD, Qingdao, 266101 China

## Abstract

H9N2 subtype avian influenza viruses (AIVs) have been isolated from various species of wild birds and domestic poultry in the world, and occasionally transmitted to humans. Although H9N2 AIVs are seldom isolated from ostriches, seven such strains were isolated from sick ostriches in China between 2013 and 2014. Sequence analysis showed several amino acid changes relating to viral adaptation in mammals were identified. The phylogenetic analyses indicated that these isolates were quadruple reassortant viruses, which are different from the early ostrich isolates from South Africa or Israel. Most of the ostrich virus carried a human-type receptor-binding property. The chicken experiments showed the ostrich strains displayed low pathogenicity, while they could cause mild to severe symptoms in chicken. Theses strains could efficiently transmit among chickens, and one strain showed higher transmissibility. The virus could not kill mice, and merely replicated in the lung of mice. The ostrich strains could not efficiently transmit between guinea pigs in the direct contact model. These results suggested we should pay attention to the interface between ostrich and other domestic fowl, and keep an eye on this population when monitoring of influenza virus.

## Introduction

Wild birds, particularly waterfowl, are the primary reservoirs of influenza A viruses (IAVs). To date, 16 and 9 different hemagglutinin (H) and neuraminidase (N) serotypes, respectively, have been identified among IAVs, and the influenza-like genomes of H17N10 and H18N11 have been screened in bats^1,2^. While avian influenza virus (AIV) usually causes an asymptomatic infection in its natural host, it can cause clinical diseases including generalized infections, upper respiratory disease and decreased egg production in aberrant hosts such as humans and domestic poultry^3,4^.

Worldwide, H5, H6, H7 and H9 IAVs have been most commonly implicated in influenza in poultry^5^. In contrast to H5N1 AIVs, H9N2 AIVs are generally considered to have low pathogenicity in chickens, so eradicating H9N2 AIVs on farms is not a priority. During the last several decades, H9N2 AIVs have been circulating widely in poultry population and caused tremendous losses, especially in China^6,7^. H9N2 AIVs can occasionally transmit to humans^8–10^. In 2013 to 2016, 18 laboratory-confirmed human cases occurred in China^11^. Additionally, H9N2 AIV donated its internal genes to the lethal H5N1 influenza virus in 1997, the H7N9 and H10N8 influenza virus in 2013, respectively^12–14^.

Ostrich husbandry is a developing industry in China, and currently the number of ostriches raised here ranks second in the world^15,16^. Ostriches are raised in open camps in most Chinese farms. Under such circumstances, ostriches readily come into contact with wild birds. Ostrich is an aberrant host for IAV, and infection with IAV can cause morbidity and mortality, especially in young birds^17^. H9N2 was first isolated from ostriches in South Africa in 1995, and subsequently in Israel in 2003, and one strain was isolated from an emu in China in 2004^17–19^. Recorded in the GenBank database (https://www.ncbi.nlm.nih.gov/genbank/) there are four H9N2 strains from ostrich to date. Consequently, there is limited knowledge about the genetic characteristics and biological properties of H9N2 influenza virus of ostrich origin. Here, we characterized the sequences, pathogenicity and transmissibility of H9N2 strains isolated from ostriches between 2013 and 2014 in China. The data from this characterization of ostrich-derived H9N2 will augment our understanding about this epidemic pathogen, and could potentially make a positive contribution to the prevention and control of H9N2 AIVs.

## Results

### Molecular characterization

To explore the genetic relationships among the seven viruses isolated from ostriches, we sequenced their whole genomes (Table 1). The HA genes of these viruses shared 95.0–99.3% sequence identity, and all of the seven viruses carried the PSRSSR↓GLF amino acid sequence at the HA cleavage site (Table 2). Six potential N-glycosylation sites (21, 128, 289, 296, 304 and 483, H3 numbering) were found in the HA protein of all seven viruses, and one potential glycosylation site was lost at position 200 in four of the viruses. A potential N-glycosylation site at position 135 was identified in one isolate (O/HB/182/14). The I155T and Q226L mutations (H3 numbering), which play important roles in H9N2 virus binding to the human-type receptor, were detected in all seven viruses^20^. The T160A, H183N, A190V mutations were found in our ostrich strains, however, no such mutations were found in the Eshkol or South African ostrich strains. HA-372K was found in our ostrich strains, in contrast, there is E/R in this site of D/HK/Y280/97 and O/RSA/9508103/95. NCBI annotated genome (http://www.ncbi.nlm.nih.gov/genomes/FLU/Database/annotation.cgi) analysis showed that the HA protein of one virus (O/YN/438/14) had a four amino acid deletion at position 220 to 223, whereas all other H9 viruses in GenBank lack this deletion. The NA genes of these viruses shared 92.1–99.9% nucleotide sequence identity, and all our ostrich strains had a 3-amino acid deletion in the NA stalk (position 63 to 65), however, such deletion was not found in other reference ostrich strains. Additionally, three viruses had potential N-glycosylation sites in the hemadsorbing (HB) site at position 366, while four viruses had a deletion of potential N-glycosylation site (position 399) in the HB site.

**Table 1:** Summary of H9N2 influenza viruses isolated from ostrich in this study.

Virus	Abbreviation	Collection date	Host	Clinic Signs	GenBank Accession number
A/ostrich/Beijing/712/2013	O/BJ/712/13	03-Mar-2013	ostrich	emaciation, pneumonedema	KP178485-KP178492
A/ostrich/Hebei/28/2013	O/HB/28/13	14-May-2013	ostrich	asthma, tracheorrhagia	KP178493-KP178500
A/ostrich/Beijing/142/2013	O/BJ/142/13	18-Jul-2013	ostrich	asthma, tracheorrhagia	KP178501-KP178508
A/ostrich/Beijing/293/2013	O/BJ/293/13	02-Nov-2013	ostrich	asthma, emaciation	KP178509-KP178516
A/ostrich/Hebei/179/2014	O/HB/179/14	19-Jul-2014	ostrich	emaciation, lung consolidation, astasia	KP178517-KP178524
A/ostrich/Hebei/182/2014	O/HB/182/14	19-Jul-2014	ostrich	emaciation, lung consolidation, astasia	KP178525-KP178532
A/ostrich/Yunnan/438/2014	O/YN/438/14	20-Dec-2014	ostrich	anorexia, emaciation, pneumonedema	KP719919-KP719926

**Table 2:** Molecular characterization of surface genes of the ostrich’s H9N2 strains.

Virus	HA^a^									NA		
	Cleavage site	Receptor binding site						Other sides		Hemadsorbing site		Deletion
		S138A	I155T	T160A	H183N	A190V	Q226L	372	220–223	366–373aa	399–404aa	in the stalk
O/BJ/712/13	RSSR↓GLF	A	T	A	N	T	L	K	RPLV	IKSDSRSG	DSDSWS	63–65
O/HB/28/13	RSSR↓GLF	A	T	A	N	A	L	K	RPLV	IKDDSRSG	DSD***NWS***	63–65
O/BJ/142/13	RSSR↓GLF	A	T	A	N	T	L	K	RPLV	IKSDSRSG	DSDSWS	63–65
O/BJ/293/13	RSSR↓GLF	A	T	A	N	V	L	K	RPLV	IRSDSRSG	DSDSWS	63–65
O/HB/179/14	RSSR↓GLF	A	T	D	N	K	L	K	RPLV	IK***NGS***RSX	DSDDWS	63–65
O/HB/182/14	RSSR↓GLF	A	T	D	N	T	L	K	RPLV	IR***NNS***RSG	DSE***NWS***	63–65
O/YN/438/14	RSSR↓GLF	A	T	A	N	V	L	K	—^b^	IR***NDS***RSG	DSE***NWS***	63–65
D/HK/Y280/97	RSSR↓GLF	A	T	A	N	T	L	R	RPLV	IKEDSRSG	DSD***NWS***	63–65
O/Eshkol/1436/03	RSKR↓GLF	A	T	S	H	E	L	K	RPLV	IKKDSRAG	DSD***NLS***	—
O/RSA/9508103/95	ASYR↓GLF	A	T	S	H	E	Q	E	RPLV	ISKDSRSG	DNN***NWS***	—

^a^H3 numbering.

^b^“—” denotes deletion.

The six internal genes of the seven H9N2 viruses showed distinct diversity, with PB2, PB1, PA, NP, M, and NS genes sharing 96.2–99.6%, 96.6–99.6%, 97.4–99.3%, 96.6–99.8%, 97.4–99.8%, and 95.4–99.7% nucleotide identity, respectively. Several amino acid changes associated with the increased replication or virulence of AIVs in mammals were detected in these H9N2 viruses (Table 3), however, these isolates do not contain E627K or D701N mutations in PB2. The following amino acid changes were observed in all our ostrich strains: L89V, G309D, R447G, and I495V in PB2^21^, I368V in PB1^22^, N383D and A515T in PA^23,24^, N30D and T215A in M1^25^, S31N in M2^26^, P42S and V149A in NS1^27–29^. In contrast, there is no PB2-I495V mutation in HK/Y280, and PB1-I368V and M2-S31N/G mutations in HK/Y280 and other reference ostrich strains.

**Table 3:** Amino acid analysis of key sites in the internal gene.

Protein	Sites	BJ/712/	HB/28/	BJ/142/	BJ/293/	HB/179/	HB/182/	YN/438/	HK/Y280	Eshkol/1436	RSA/9508103	Function^a^
PB2	L89V	V	V	V	V	V	V	V	V	V	V	Increased virulence in mice^21^
	G309D	D	D	D	G	D	D	D	D	D	D	
	R477G	G	G	G	G	G	G	G	G	G	G	
	I495V	V	V	V	V	V	V	V	T	V	V	
	E627K	E	E	E	E	E	E	E	E	E	E	Increased virulence and transmissibility in mammals^46,47^
	D701N	D	D	D	D	D	D	D	D	D	D	Increased virulence and transmissibility in mammals^46,47^
PB1	L13P	P	P	P	P	P	P	P	P	P	uk	Mammalian host-specific markers^43^
	I368V	V	V	V	V	V	V	V	I	I	I	Airborne transmissibility in ferrets^22^
PA	N383D	D	D	D	D	D	D	D	D	D	D	Increased virulence in ducks^23^
	A515T	T	T	T	T	T	T	T	T	T	T	Increased virulence in ducks^24^
	F672L	L	L	L	L	L	L	L	L	L	L	Airborne transmissibility in chickens^44^
M1	V15I	I	I	I	I	I	I	I	I	I	V	Mammalian host-specific markers^9^
	N30D	D	D	D	D	D	D	D	D	D	D	Increased virulence in mice^25^
	T215A	A	A	A	A	A	A	A	A	A	A	Increased virulence in mice^25^
M2	S31N/G	N	N	N	N	N	N	N	S	S	S	Resistance to M2-blocker antiviral drugs^48^
	L55F	F	F	F	F	F	F	F	F	F	L	Mammalian host-specific markers^9^
NS1	P42S	S	S	S	S	S	S	S	S	S	S	Increased virulence in mice^28^
	V149A	A	A	A	A	A	A	A	A	A	A	Increased virulence in mice^29^

^a^The functions of each site were described in brief, and the related references were superscript.

### Phylogenetic relationships

**Figure 1:**
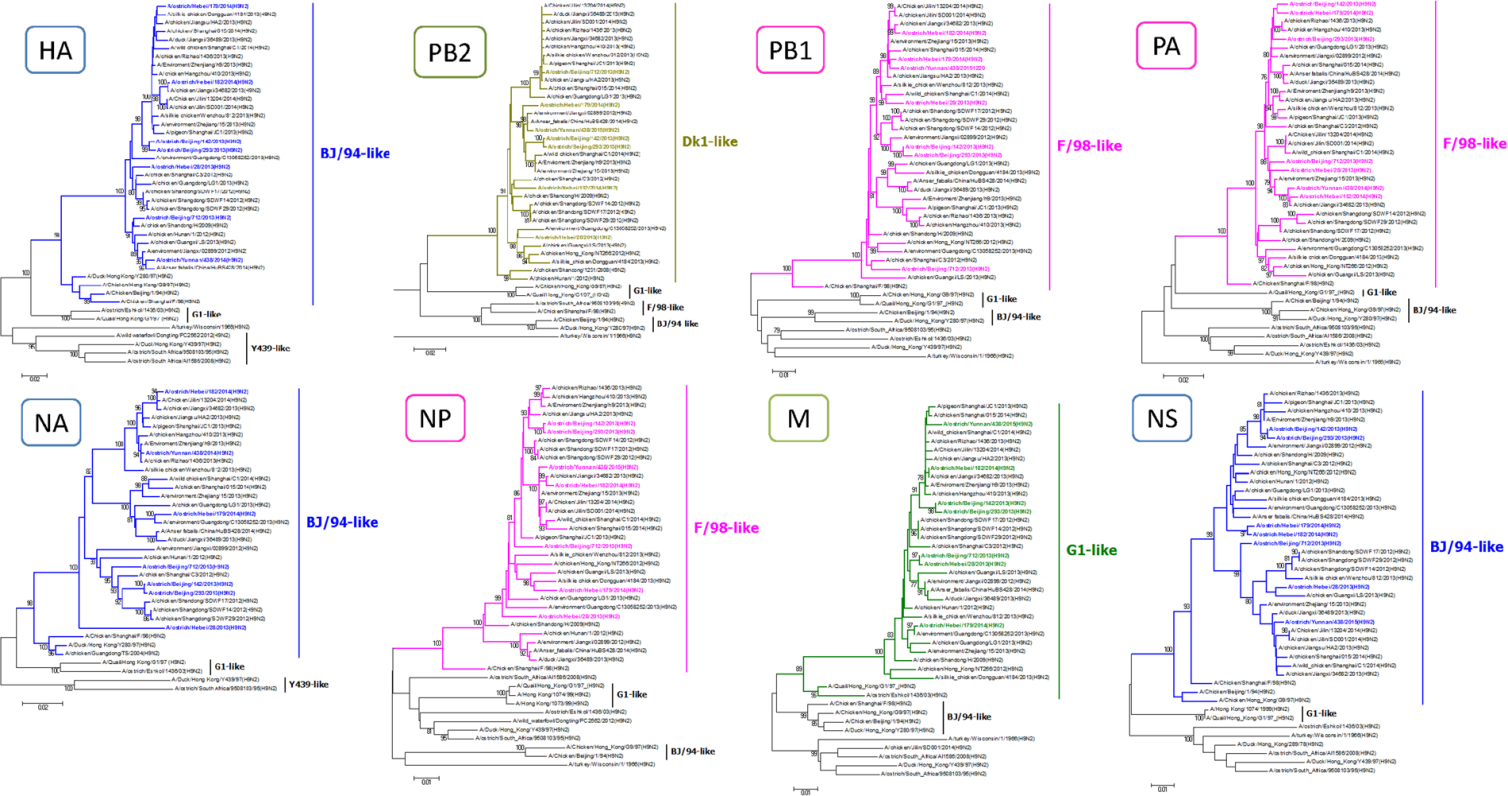
Genetic relationships among the ostrich and other H9N2 strains. Phylogenetic trees of HA (**A**), NA (**B**), PB2 (**C**), PB1(**D**), PA (**E**), NP (**F**), M (**G**) and NS (**H**) genes of H9N2 viruses was constructed by MEGA using neighbor-joining method. The ostrich H9N2 isolates and identified cluster are highlighted in colors. Supporting bootstrap values of greater than 75 are shown.

The phylogenetic relationships of all eight genes for the seven ostrich viruses were compared with the reference sequences obtained from GenBank. The phylogenetic analysis based on the genome showed that the seven viruses were quadruple reassortants (Fig. 1), with HA, NA and NS gene belonging to BJ/94-like (A/chicken/Beijing/1/1994) sublineage, PB2 and M gene originating from the prototype Dk1(A/duck/Jiangsu/1/2008) and G1(A/Quail/Hong Kong/G1/1997) respectively, and PB1, PA and NP gene deriving from F98 virus (A/chicken/Shanghai/F/1998). However, the ostrich H9N2 viruses isolated in South Africa and Israel had close relationship with Y439 virus (A/duck/Hong Kong/Y439/97), or G1 virus, indicating that these new isolates differ from the early viruses collected from ostriches. Interestingly, unlike the H9N2 viruses circulating in 2012 to 2015 in China, our ostrich H9N2 viruses failed to form an independent branch on the tree. Basic Local Alignment Search Tool (BLAST, https://blast.ncbi.nlm.nih.gov/Blast.cgi) analysis of the gene segments in GenBank found that the most similar viruses were C/SD/qd0516/12 (H9N2) and C/HB/0721/13 (H9N2), etc. (Table S1).

### Receptor binding properties

**Figure 2:**
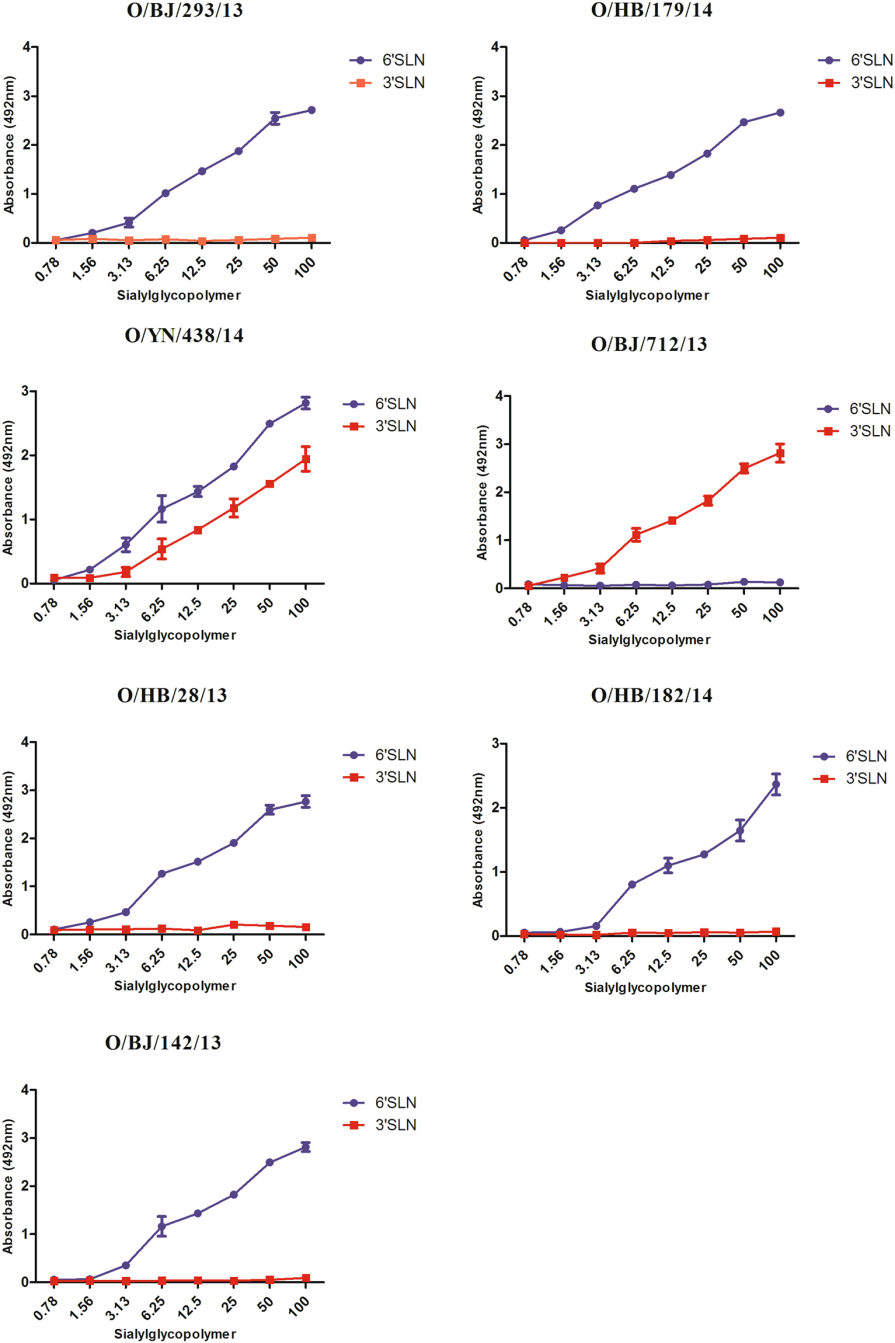
Characterization of the receptor-binding specificity of the ostrich H9N2 viruses. The analysis of receptor-binding specificities of the influenza virus was performed using a direct solid-phase assay. Each value presented is the mean ± SD of three experiments, which were each performed in triplicate.

To test the receptor binding properties of the ostrich virus, a solid-phase direct binding assay was used. The results showed most of ostrich virus only bond to the α2, 6 glycan, and carried an human-type receptor- binding property. However, O/BJ/712/13 only bond to α2, 3 glycan, showed an avian-type receptor-binding property. O/YN/438/14 bond to both α2, 3 and α2, 6 glycan, showed dual receptor-binding property (Fig. 2).

### Pathogenicity in chickens

In order to test the pathogenicity of the ostrich H9N2 viruses, Thirty four-week-old SPF chickens were infected and observed for ten days post-infection (dpi). In the O/BJ/293/13 virus-infected group, one chicken showed severe clinical signs at 4 dpi and died at 5 dpi. Nine chickens showed mild clinical signs from 2 to 7 dpi, of which seven gradually recovered to normality from 7 dpi. The remaining two chickens still showed mild clinical signs until the end of the observation period. In the O/YN/438/14 virus-infected group, the disease signs and disease course were similar to those of the O/BJ/293/13 virus-infected group, but there were no fatalities. The IVPI value was 0.63 for the O/BJ/293/13 virus, and 0.4 for O/YN/438/14 virus (Table S2). The O/BJ/293/13 virus was detected in the lung of chickens euthanized at 3 and 5 dpi, while the O/YN/438/14 virus was only detected in the lung of chickens euthanized at 5 dpi. Chicken inoculated with O/BJ/293/13 had a higher virus titer (2.5, 2.5 and 3.0 log_10_EID_50_/ml) than those inoculated with O/YN/438/14 (0, 2.25 and 2.5 log_10_EID_50_/ml) at 5 dpi.

### Virus transmission among chickens

Twenty-four five-week-old SPF chickens were infected with O/BJ/293/13 or O/YN/438/14 virus (Table 4). In the direct inoculation group (O/BJ/293/13), the virus was detected in the tracheae swab samples from 1 to 7 dpi, and in the cloacae swab samples from 3 to 5 dpi. In the O/BJ/293/13 physical contact group, the virus was detected in the tracheae swab samples from 3 to 9 dpi, and in the cloacae swab samples from 3 to 5 dpi. In contrast, in the O/YN/438/14 direct inoculation group, the virus was detected in the tracheae swab samples from 1 to 9 dpi, and in the cloacae swab samples from 3 to 5 dpi. In the O/YN/438/14 physical contact virus group, the virus was detected in the tracheae swab samples from 7 to 9 dpi, and no virus was detected in the cloacae swab samples.

**Table 4:** Transmission test in chickens.

dpi	O/BJ/293/13				O/YN/438/14			
	Direct inoculation		Physical contact		Direct inoculation		Physical contact	
	Trachea	Cloaca	Trachea	Cloaca	Trachea	Cloaca	Trachea	Cloaca
1	3/3	0/3	0/3	0/3	2/3	0/3	0/3	0/3
3	3/3	3/3	3/3	2/3	3/3	2/3	0/3	0/3
5	3/3	1/3	2/3	1/3	3/3	1/3	0/3	0/3
7	3/3	0/3	2/3	0/3	2/3	0/3	2/3	0/3
9	0/3	0/3	2/3	0/3	1/3	0/3	1/3	0/3
11	0/3	0/3	0/3	0/3	0/3	0/3	0/3	0/3

Five-week-old SPF chickens were divided into the direct inoculation group and physical contact group for each virus (O/BJ/293/13 and O/YN/438/14). The physical contact group was raised in the same cage as the chickens from the inoculation group, from 24 hours post-inoculation. The direct inoculation group was inoculated intranasally or intratracheally with 10^7.0^ EID_50_/mL of virus. The chickens in direct inoculation group and the physical contact group were swabbed every other day in their tracheae and cloacae from 1 to 15 dpi using 1 mL of isolation media (50% glycerol in PBS, 0.5% gentamycin, and 1% mycostatin). The viruses recovered were titrated for infectivity in 10-day-old embryonated chicken eggs.

### Pathogenicity in mice

**Figure 3:**
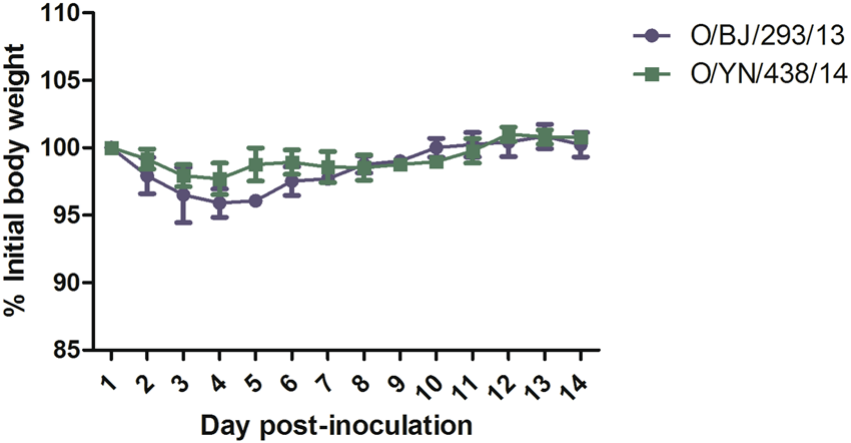
Mean changes in body weight of mice infected with H9N2 viruses. BALB/c mice were inoculated i.n. with the ostrich H9N2 viruses at a dose of 10^6^ EID_50_. The body weights were monitored daily for a 14-day observation period and expressed as a percentage of the initial value. The data represents the mean ± SD of five mice in each group.

To test the pathogenicity of the ostrich virus in mammal model, groups of mice were inoculated with O/BJ/293/13 and O/YN/438/14 virus. The two group mice showed a slight loss of body weight between 1 dpi and 4 dpi, and then gradually gained body weight (Fig. 3). No obvious signs could be found, except loss of weight and inactivity. The infectivity and replication ability of the ostrich virus in mice was evaluated by the virus titers in the lung and other organs of the infected mice at 3 and 5 dpi. The results indicated the two viruses could infect and replicate in mouse lung without prior adaptation, and mice inoculated with O/BJ/293/13 had a higher virus titer than those inoculated with O/YN/438/14. No virus could be detected in other organs (Table 5).

**Table 5:** Replication of H9N2 influenza viruses in mice^a^.

	O/BJ/293/13					O/YN/438/14				
	lung	heart	brain	spleen	liver	Lung	heart	brain	spleen	liver
3 dpi	3.2 ± 0.6 (3/3)^b^	—	—	—	—	2.1(1/3)	—	—	—	—
5 dpi	2.3(1/3)	—	—	—	—	—	—	—	—	—

^a^Six-week-old female BALB/c mice were inoculated intranasally with 10^6^ EID_50_ of H9N2 influenza viruses. Three mice from each group were killed on 3 dpi and 5 dpi and virus titers in samples of heart, liver, spleen, lung and brain were determined. ^b^The average virus titer was expressed as log_10_EID_50_/ml ± SD(positive number/total number), with the limit of virus detection at 1.2 log_10_ EID_50_/ml. ^c^— indicates that virus was not recovered from the sample.

### Virus transmission among guinea pigs

**Figure 4:**
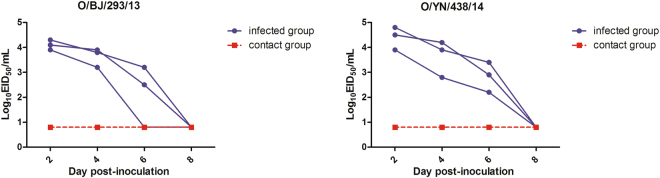
Assessment of direct contact-mediated transmissibility of the ostrich H9N2 AIVs in guinea pigs. Groups of three SPF guinea pigs were intranasally with 10^6^ EID_50_ of tested virus and housed in a cage. At 24 h post-inoculation, three naïve guinea pigs were placed in the same cage and co-housed. At 2, 4, 6 and 8 dpi, the nasal washes of the three inoculated animals and three contact animals were collected and tittered by EID_50_ assay. Each line represents the virus titer from an individual animal. The dashed lines indicate the limit of virus detection.

To investigate the transmissibility of the ostrich virus in mammals, groups of three guinea pigs were inoculated i.n. with 10^6^ EID_50_ of O/BJ/293/13 and O/YN/438/14 virus, after 24 h post-inoculation, three naïve guinea pigs were placed in the same cage and co-housed. To assess productive transmission, nasal washes collected from the inoculated and contact guinea pigs at various time points post-contact for the presence of virus were tested and serum for HI antibody responses after 21 days were assessed. The results showed while virus was detected in the nasal washes of all inoculated guinea pigs following i.n. inoculation with each H9N2 virus from 2 to 4 dpi, none of the naive contact guinea pigs seroconverted or had detectable virus in nasal washes (Fig. 4).

## Discussion

H9N2 AIVs have been circulating widely in the poultry population, where they cause a mild respiratory disease and reduction in egg production in many Asian countries over the last several decades^20^. In GenBank, there are more than 4100 records of H9N2 IAVs. The host ranges of H9N2 IAVs include domestic poultry, wild birds, swine, canines, horses, mink and humans. Ostrich is a species of large flightless bird native to Africa, and ostrich farming originated in South Africa in the 1860s. IVAs can infect domestic and wild ostriches, and cause disease in them. Influenza subtypes H3, H4, H5, H6, H7, H9 and H10 have been detected in ostriches, rheas and emus^30–32^. Between 2013 and 2014, several outbreaks of H9N2 influenza occurred in ostrich farms and resulted in significant economic losses in China. Despite the pathogenic and molecular characters of chicken-derived H9N2 AIVs being regularly reported, H9N2 AIV strains from ostriches are poorly characterized. The present work analyzed the genetic character of H9N2 viruses isolated from ostriches, and explored their biological characteristics.

Phylogenetic analysis showed that the seven isolates belonged to the same sublineage in all eight gene segment phylogenetic trees, reflecting the low genetic diversity of H9N2 AIVs circulating in ostriches. At present, most ostriches breed in an open field, where they can easily make contact with poultry or wild birds. The BLAST results from GenBank showed that the seven isolates had high homology with the H9N2 viruses circulating among domestic fowl in China, indicating these ostrich H9N2 viruses were probably transmitted from domestic fowl.

Sequence analysis showed all seven viruses carried the amino acid sequence PSRSSR↓GLF at the HA cleavage site, which was tipical of low-pathogenic AIV^33–35^, and was confirmed by the chicken intravenous pathogenicity test. The HA glycoprotein is a major determinant of host switching, primarily because of its role in host cell receptor recognition^27,36^. All seven viruses carry the Q226L mutation in the receptor-binding pocket of HA1, which is related to its binding ability to α-2, 6-linked sialic acid receptors^37–39^. Furthermore, five other amino acid changes (S138A, I155T, T160A, H183N, and A190V, H3 numbering) in HA, which are known to affect the receptor binding preference of influenza virus, were also detected in these viruses^20,40^. In contrast, no T160A, H183N, A190V mutations were found in the early ostrich strains, indicating the receptor-binding property may differ from our strains. A potential N-glycosylation site at position 135 was observed in one isolate, which is supposed to affect virus-induced cell fusion and its receptor binding ability^41^. The deletion of HA-220aa to 223aa of O/YN/438/14 is uncommon, however, the animal experiments indicate these deletion may not significantly influence its virulence and transmissibility in chicken and mammals.

The solid-phase binding assay results showed most of the ostrich viruses carried a human-type receptor-binding property. These results may be attributed to the Q226L and other key mutations affecting receptor-binding. However, O/BJ/712/13 with the Q226L mutation, showed a single avian-type receptor-binding property, indicating other residues may influence the function of Q226L and other function site. The transmission experiment showed these ostrich viruses couldn’t efficiently transmit among guinea pigs through direct contact. The phenomenon indicates the transmission ability is not just due to receptor-binding properties. A previous study showed H9N2 AIVs could obtain efficient transmission ability through direct contact, after 15 serial passages in guinea pigs. Host adaptation needs a long-term process, and several genes relate to the transmissibility.

All the seven viruses identified herein have a 3-amino acid deletion in the NA stalk, and this deletion has been shown to increase HA cleavage efficiency when combined with an alanine-to-serine (A → S) substitution at the P5 cleavage site (A325S, H3 numbering)^42^. Sequence analysis of the internal gene products also identified mammalian host-specific markers in M (M1 V15I; M2 L55F) and PB1 (L13P)^9,43^. The seven viruses contain HA and PA genes with HA-372K (H3 numbering) and PA-627L, respectively, which are both important for airborne transmissibility among chickens^44^, however, other reference ostrich strains do not contain these properties. A previous study on H5N1 virus infection in ostriches showed that ostrich cells facilitated the emergence of viruses possessing mammalian-type PB2-627K and/or PB2-701N^45^. In this study, no PB2-627K or PB2-701N mutation was found in these viruses^46,47^, indicating that these mammalian-type mutations in ostriches may have subtype specificity. In addition, S31N in M2 was detected in all seven viruses, suggesting resistance to M2-blocker antiviral drugs such as amantadine^48^, in contrast, no such mutation was found in HK/Y280 or other reference ostrich strains.

The transmission experiment in chickens revealed that the ostrich viruses could efficiently transmit between chickens, although they carried a human-type receptor-binding property. The mouse study showed these ostrich H9N2 strains could not kill mice with a high inoculation dosage, and merely replicated in mouse lung. Although some mammalian receptor-binding and host-specific mutations were found in these ostrich strains, they didn’t fundamentally change the infectivity and pathogenicity to mammals. These H9N2 viruses were isolated from ill ostrich, and most of the infected ostrich showed respiratory symptoms, which is similar to the clinic signs of H9N2 influenza in chicken. A limitation of our study is that we could not conduct infection tests in ostriches because of the lack of animal testing facilities.

In summary, we have reported the genetic and phylogenetic characterization of seven H9N2 AIVs isolated from ill ostriches. Phylogenetic analyses showed the seven H9N2 AIVs were quadruple reassortant viruses, and different from the South African and Israeli isolates. Several amino acid changes related to the increased replication or virulence of avian influenza viruses in mammals were identified in these H9N2 viruses. These ostrich H9N2 AIVs had low pathogenicity in chickens, but showed high transmissibility among them.

## Materials and Methods

### Ethics Statement

The study was carried out in strict accordance with the recommendations in the Guide for the Care and Use of Laboratory Animals of the Ministry of Science and Technology of the People’s Republic of China. The protocols for the animal studies were approved by College of Animal Science and Technology of Qingdao Agricultural University.

### Biological facility

All experiments with live H9N2 viruses were conducted within the enhanced animal biosafety level 2+ (ABSL2+) facilities in Qingdao Oland-Better Bioengineering Co., LTD. The animal isolators in the facility are HEPA filtered.

### Clinical samples

Tissue samples (lung, spleen, liver, heart, and brain), synovial fluid, tracheal and cloacal swabs were obtained from ill ostriches submitted to the Chinese Center for Ostrich Disease Prevention and Control (Qingdao Agriculture University) (from Mar. 2013 to Dec. 2014). The clinical signs observed in the ill ostriches included depression, emaciation, respiratory symptoms and etc. (Table 1).

### Virus isolation and identification

For virus isolation, tracheal swabs were taken and placed in 1.0 mL of transmission medium [50% (vol/vol) glycerol in phosphate-buffered saline, PBS] containing antibiotics, as described previously^49^. Tissues were homogenized in the PBS-antibiotic solution. Samples were centrifuged at 1,000 × g for 10 min at 4 °C, and 0.2 mL of each supernatant was inoculated into the allantoic cavity of individual 10-day-old special pathogen-free (SPF) embryonated chicken eggs, after which the eggs were incubated for 48–96 h at 37 °C with daily monitoring of each embryo. Allantoic fluid was harvested and tested for hemagglutinin (HA) activity, and the HA-positive samples were identified serologically with antisera to each known influenza virus subtype, as described previously^50^. H9N2 virus-containing allantoic fluid was harvested and stored as stock at −80 °C for viral RNA extraction and the animal experiments. The 50% egg infectious dose (EID_50_) was determined by serial dilutions of the virus in eggs and the value was calculated using the method described by Reed and Muench^51^.

### RNA isolation and amplification

Viral RNA was extracted from allantoic fluid with RNAiso Plus reagent using an RNA extraction kit in accordance with the manufacturer’s instructions (TaKaRa, TaKaRa Biotechnology Co. Ltd., Dalian, China). The RNA was then used to amplify all the complete fragments of AIV by reverse-transcriptase polymerase chain reaction (RT-PCR) using the One Step RT-PCR Kit (TaKaRa) with eight primer pairs, as reported previously^52^.

### Genomic sequencing

The full genomes of the seven H9N2 AIVs were sequenced by Sanger sequencing on an ABI 3730 genetic analyzer (Applied Biosystems, Carlsbad, CA, USA). DNA sequences for all the genes were edited, compiled, assembled, and analyzed using SeqMan in Lasergene 7 (DNASTAR, Madison, WI, USA). The genomic sequences of the seven H9N2 AIVs are available in GenBank under the accession numbers KP178485–KP178516, KP178517–KP178524, KP178525– KP178532 and KP719919–KP719926.

### Phylogenetic analysis and molecular characterization

The reference sequences from the Influenza Virus Resource at the National Center for Biotechnology Information (NCBI) (www.ncbi.nlm.nih.gov/genomes/FLU) and the Global Initiative on Sharing Avian Influenza Data (www.gisaid.org) were downloaded. The phylogenetic trees for the isolates were constructed by MEGA 6.05 using the neighbor-joining method with 1000 bootstrap replicates. MegAlign in Lasergene 7 (DNASTAR) was used to determine the nucleotide sequence similarity percentages, and the NetNGlyc 1.0 Server (http://www.cbs.dtu.dk/services/NetNGlyc/) was used to predict the glycosylation sites in the HA and neuraminidase (NA) genes.

### Solid-phase binding assay

The analysis of receptor-binding specificities of the influenza virus was performed using a direct solid-phase assay. In brief, microtiter plates (Pierce, USA) were incubated with two different glycopolymers: (Neu5Acα 2-3Galβ 1-4GLcNAcβ 1-4GlcNAcβ -PAA-biotin and Neu5Acα 2-6Galβ 1-4GLc NAcβ 1-4GlcNAcβ -PAA-biotin; GlycoTech Corporation, USA) at 4 °C overnight. After removing the glycopolymer solution, the plates were blocked with 0.1 ml of PBS containing 2% bovine serum albumin (Invitrogen) at room temperature for 1 h. The plates were washed with PBS four times and then incubated in a solution containing influenza virus (64 hemagglutination units in PBS) at 4 °C over night. After four washes with PBST (PBS containing 0.1% Twen-20), an antibody against H9 influenza virus was added to the plates and the plates were incubated at 4 °C for five additional hours. The plates were then washed three times with PBST and incubated with the horseradish peroxidase(HRP)-conjugated goat-anti-chicken antibody (Sigma-Aldrich, St. Louis, MO, USA) for two hours at 4 °C.The plates were then washed five times and incubated with O-phenylenediamine (Sigma-Aldrich, St. Louis, MO, USA) in PBS containing 0.01% H_2_O_2_ for 10 min at room temperature. The reaction was stopped with 0.05 ml of 0.5 M H_2_SO_4_.The absorbance was determined at 490 nm.

### Chicken intravenous pathogenicity tests

Two ostrich H9N2 viruses (O/BJ/293/13 and O/YN/438/14) were selected for pathogenicity and transmission tests, based on their phylogenic relationships and spatial distributions with other H9N2 viruses. The tests were performed in accordance with the instructions in the World Organization for Animal Health (OIE) manual. Four-week-old SPF Leghorn chickens (n = 10 per group, Beijing Merial Vital Laboratory Animal Technology Co., Ltd, Beijing, China) were intravenously inoculated with 0.1 mL of a 1:10 dilution of allantoic fluid, and the control group was inoculated with PBS. The intravenous pathogenicity index (IVPI) for the virus in chickens was determined according to OIE recommendations. The chickens were housed in stainless steel isolation cabinets that were ventilated under negative pressure with HEPA-filtered air, and care was provided as required.

### Transmission experiments in chickens

For the experiments, twelve SPF White Leghorn chickens (5-week-old) were divided into the direct inoculation group (nine chickens) and physical contact group (three chickens) for each virus (O/BJ/293/13 and O/YN/438/14). The physical contact group was raised in the same cage as the chickens from the inoculation group, from 24 hours post-inoculation. The direct inoculation group was inoculated intranasally or intratracheally with 10^7.0^ EID_50_/mL of virus. One milliliter of virus inoculum was administered to the chickens as follows: 0.2 mL for the intranasal inoculations, and 0.8 mL for the tracheal inoculations. Three chickens from the direct inoculation group were euthanized at 3 and 5 days post-infection; their lungs were collected and weighed, then homogenized using a Retsch Tissue Lyser MM400 machine (30 cycles/s, 4 min) in cold PBS under sterile conditions. Next, the solid debris was pelleted by centrifugation at 5,000 × g for 10 min, and the homogenates were used for virus titrations in 10-day-old embryonated chicken eggs. The remaining chickens from the direct inoculation group and the physical contact group were swabbed every other day in their tracheae and cloacae from 1 to 15 dpi using 1 mL of isolation media (50% glycerol in PBS, 0.5% gentamycin, and 1% mycostatin). The viruses recovered were titrated for infectivity in 10-day-old embryonated chicken eggs.

### Mouse experiments

Groups of 5-week-old female BALB/c mice (Beijing Experimental Animal Center) were anesthetized with Zoletil 50 (tiletamine- zolazepam; Virbac S.A., Carros, France; 20 μg/g) and inoculated intranasally with 10^6^ 50% egg infectious doses (EID_50_) of viruses in 50 μl phosphate buffered saline (PBS). Three mice in each group were euthanized at 3 and 5 dpi, lungs, hearts, livers, spleens and brains were collected for virus titration in eggs. The remaining five mice in each group were monitored for weight loss and mortality for 14 days. Mice that lost more than 25% of their body weight were humanely euthanized.

### Contact transmission in guinea pigs

Groups of three SPF guinea pigs weighing 300–350 g were intranasally with 10^6^ EID_50_ of tested virus and housed in a cage. At 24 h post-inoculation, three naïve guinea pigs were placed in the same cage and co-housed. At 2, 4, 6 and 8 dpi, the nasal washes of the three inoculated animals and three contact animals were collected and tittered by EID_50_ assay. All of the guinea pigs were humanely euthanized at 21 dpi and tested for seroconversion.

### Data availability

All data generated or analysed during this study are included in this published article (and its Supplementary Information files).

## Electronic supplementary material

Table S1 and S2
