# Supplementary material for: Characterization of avian influenza H9N2 viruses isolated from ostriches (Struthio camelus)
Source: Sci Rep. 2018 Feb 2;8:2273. doi: 10.1038/s41598-018-20645-1 (PMC5797180; doi:10.1038/s41598-018-20645-1)
Supplement: Supplementary file 1 — Table S1 and S2 [file 41598_2018_20645_MOESM1_ESM.pdf]

Characterization of avian influenza H9N2 viruses isolated from ostriches (*Struthio camelus*)

Dongdong Wang[a*], Jingjing Wang[a,b*], Yuhai Bi[c], Dandan Fan[a], Hong Liu[a], Ning Luo[a], Zongtong Yang[a], Shouchun Wang[a], Wenya Chen[d], Jianlin Wang[a], Shouzhen Xu[a], Jiming Chen[b], Yi Zhang[b], Yanbo Yin[a]

[a]Laboratory of Preventive Veterinary Medicine, College of Animal Science and Veterinary Medicine, Qingdao Agricultural University, Qingdao 266019, China.

[b]China Animal Health and Epidemiology Center, Qingdao 266032, China.

[c]CAS Key Laboratory of Pathogenic Microbiology and Immunology, Institute of Microbiology, Center for Influenza Research and Early-warning (CASCIRE), Chinese Academy of Sciences, Beijing, China

[d]Qingdao Oland-Better Bioengineering Co., LTD., Qingdao 266101, China.

Correspondence:

Yanbo Yin, yanboyin2011@163.com.

Yi Zhang, zhangyi@cahec.cn

Table S1. Similarity scores for individual genes from 7 H9N2 strains from ostrich and the corresponding genes in GenBank.

Virus	PB2	PB1	PA	HA	NP	NA	M	NS
O/BJ/712/13	C/BJ/1115/13(H9N2)[a] [KM609875.1] 98%	C/SD/qd0516/12(H9N2) [KM609815.1] 99%	C/SD/qd0516/12(H9N2) [KM609775.1] 99%	C/SD/qd0516/12(H9N2) [KM609575.1] 100%	C/SD/qd0516/12(H9N2) [KM609695.1] 99%	C/SD/qd0516/12(H9N2) [KM609655.1] 99%	C/TJ/614/12(H9N2) [KF059287] 99%	C/WX/04030201G/13/(H7N9) [KR905379.1] 98%
O/HB/28/13	C/SD/wf0514/13(H9N2) [KM609847.1] 98%	C/SD/wf0514/13(H9N2) [KM609807.1] 99%	C/SD/wf0514/13(H9N2) [KM609767.1] 100%	C/SD/wf0514/13(H9N2) [KM609567.1] 99%	C/SD/wf0514/13(H9N2) [KM609687.1] 99%	C/SD/wf0514/13(H9N2) [KM609647.1] 99%	C/SD/wf0514/13(H9N2) [KM609607.1] 99%	C/SZ/040207GH05/13/(H7N9) [KR905370.1] 99%
O/BJ/142/13	C/HB/0721/13(H9N2) [KM609872.1] 99%	C/HB/0721/13(H9N2) [KM609832.1] 99%	C/HB/0721/13(H9N2) [KM609792.1] 100%	C/HB/0721/13(H9N2) [KM609592.1] 99%	C/HB/0721/13(H9N2) [KM609712.1] 99%	C/HB/0721/13(H9N2) [KM609672.1] 99%	C/HB/0721/13(H9N2) [KM609632.1] 99%	C/SD/qd01417/13(H9N2) [KM609740.1] 100%
O/BJ/293/13	C/HB/0721/13(H9N2) [KM609872.1] 99%	C/HB/0721/13(H9N2) [KM609832.1] 99%	C/JS/WJ57/12(H9N2) [KJ000709.1] 99%	C/HB/0721/13(H9N2) [KM609592.1] 99%	C/HB/0721/13(H9N2) [KM609712.1] 99%	C/HB/0721/13(H9N2) [KM609672.1] 99%	C/HB/0721/13(H9N2) [KM609632.1] 99%	C/SD/qd01417/13(H9N2) [KM609740.1] 99%
O/HB/179/14	C/SD/wf0514/13(H9N2) [KM609847.1] 98%	D/AH/SC702/13(H7N9) [CY147058.1] 99%	C/JS/XZ0616/12(H9N2) [KJ426375.1] 99%	C/ST/4329/14(mixed) [KP418192.1] 99%	D/GD/C3204/10(H9N2) [KM113166.1] 98%	C/SZ/515/13/(H9N2) [KP414262.1] 99%	C/SD/yt0106/12(H9N2) [KM609603] 99%	C/GD/01/11(H9N2) [KC8821273] 99%
O/HB/182/14	C/SD/qd0516/12(H9N2) [KM609855.1] 98%	C/YT/2468/13(H9N2) [KP414700.1] 99%	C/YT/2529/13(mixed) [KP415204.1] 99%	C/YT/2468/13(H9N2) [KP414702.1] 99%	C/YT/2243/13(H9N2) [KP415180.1] 99%	C/YT/2243/13(H9N2) [KP4151801.1] 99%	C/BJ/0331/13(H9N2) [609637..1] 99%	C/GD/01/11(H9N2) [KC8821273] 99%
O/YN/438/14	C/RZ/2658/13(H9N2) [KP415216.1] 99%	G/JS/1027/13(H7N9) [KP455976.1] 99%	D/SN/04802G/13(H7N9) [KM879372.1] 99%	C/SD/TA05/13(H9N2) [KP006605.1] 99%	H/AH/1-YK RG202/13(H7N9) [CY193254.1] 99%	C/RZ/3052/13(H9N2) [KP4144792.1] 100%	G/JS/1027/13(H7N9) [KP455981.1] 99%	C/WX/04030201G/13(H7N9) [KR905379.1] 99%

[a]The accession numbers and similarity scores are shown after the strain name.

Table S2.1. Clinical data for the chicken intravenous pathogenicity test for O/BJ/293/13.

Clinical symptoms	1 d.p.i.	2 d.p.i.	3 d.p.i.	4 d.p.i.	5 d.p.i.	6 d.p.i.	7 d.p.i.	8 d.p.i.	9 d.p.i.	10 d.p.i.	Total	Value
Normal (0)	10	8	4	3	1	0	3	7	7	7	50	0
Mild symptoms (1)	0	2	6	6	8	9	6	2	2	2	43	43
Severe symptoms (2)	0	0	0	1	0	0	0	0	0	0	1	2
Death (3)	0	0	0	0	1	1	1	1	1	1	6	18
IVPI Value												0.63

Table S2.2. Clinical data for the chicken intravenous pathogenicity test for O/YN/438/14.

Clinical symptoms	1 d.p.i.	2 d.p.i.	3 d.p.i.	4 d.p.i.	5 d.p.i.	6 d.p.i.	7 d.p.i.	8 d.p.i.	9 d.p.i.	10 d.p.i.	Total	Value
Normal (0)	10	8	7	5	3	2	4	7	7	7	60	0
Mild symptoms (1)	0	2	3	5	7	8	6	3	3	3	40	40
Severe symptoms (2)	0	0	0	0	0	0	0	0	0	0	0	0
Death (3)	0	0	0	0	0	0	0	0	0	0	0	0
IVPI Value												0.4

Table S2.3. Clinical data for the chicken intravenous pathogenicity test for PBS.

Clinical symptoms	1 d.p.i.	2 d.p.i.	3 d.p.i.	4 d.p.i.	5 d.p.i.	6 d.p.i.	7 d.p.i.	8 d.p.i.	9 d.p.i.	10 d.p.i.	Total	Value
Normal (0)	10	10	10	10	10	10	10	10	10	10	100	0
Mild symptoms (1)	0	0	0	0	0	0	0	0	0	0	0	0
Severe symptoms (2)	0	0	0	0	0	0	0	0	0	0	0	0
Death (3)	0	0	0	0	0	0	0	0	0	0	0	0
IVPI Value												0

The tests were performed in accordance with the instructions in the World Organization for Animal Health (OIE) manual. Four-week-old SPF

Leghorn chickens (n=10 per group, Beijing Merial Vital Laboratory Animal Technology Co., Ltd, Beijing, China) were intravenously inoculated with 0.1 mL of a 1:10 dilution of allantoic fluid, and the control group was inoculated with PBS. The intravenous pathogenicity index (IVPI) for the virus in chickens was determined according to OIE recommendations.
